# Circulating tumor DNA in colorectal cancer: assay selection, clinical applications, and practical integration for gastroenterologists

**DOI:** 10.3389/fonc.2026.1894198

**Published:** 2026-08-03

**Authors:** Ashish Sharma, Joecelyn Kirani Tan, Harendra Kumar, Arkadeep Dhali, Ruchir Paladiya, Rajvardhan Sisodia, Hareesha Bharadwaj, Dushyant S. Dahiya, Saikat Mandal

**Affiliations:** 1 Department of Internal Medicine, Yale New Haven Hospital, New Haven, CT, United States; 2 Faculty of Biology, The University of Manchester, Manchester, United Kingdom; 3 Department of Internal Medicine, Mayo Clinic, Rochester, MN, United States; 4 Sheffield Teaching Hospitals NHS Foundation Trust, Sheffield, United Kingdom; 5 University of Connecticut School of Medicine, Farmington, United States; 6 Mahatma Gandhi Memorial Medical College, Indore, India; 7 Royal Stoke University Hospital, Stoke-on-Trent, United Kingdom; 8 The University of Kansas School of Medicine, Kansas City, KS, United States; 9 University of Nottingham School of Medicine, Nottingham, United Kingdom; 10 NIHR Nottingham Biomedical Research Centre, Nottingham University Hospitals NHS Trust and University of Nottingham, Nottingham, United Kingdom

**Keywords:** adjuvant chemotherapy, biomarker-guided therapy, circulating tumor DNA, clonal hematopoiesis, colorectal cancer, liquid biopsy, microsatellite instability, minimal residual disease

## Abstract

**Background:**

Circulating tumor DNA (ctDNA) has rapidly evolved from a research tool to a clinically applicable biomarker in colorectal cancer (CRC), transforming the assessment of minimal residual disease (MRD) and recurrence risk. The growing availability of commercial assays has complicated test selection and interpretation, particularly for gastroenterologists who increasingly encounter ctDNA data in postoperative and surveillance settings.

**Methods:**

We conducted a narrative review of the literature using PubMed, Embase, and Cochrane databases from January 2015 through March 2026, prioritizing randomized controlled trials, prospective cohort studies, and clinical practice guidelines relevant to ctDNA applications in CRC. The search employed predefined keyword combinations applied independently to each database. Two authors (A.S. and S.M.) independently screened titles and abstracts against the prespecified inclusion criteria, with disagreements resolved by discussion.

**Results:**

Tumor-informed assays that analyze patient-specific mutations exhibit high specificity for MRD detection, with serial sampling sensitivity reaching 88% compared to 59% for tumor-agnostic approaches, based on a 2026 systematic review and diagnostic accuracy meta-analysis that pooled data across multiple platforms and settings; the authors note substantial study-level heterogeneity in platform design and patient populations, and results should be interpreted accordingly, but require tissue access and extended processing times. Tumor-agnostic tests are more rapid and accessible, incorporating genomic and epigenomic signals, though they exhibit site-specific sensitivity variations ranging from 100% for liver metastases to 40% for peritoneal recurrence and remain vulnerable to biological confounders such as clonal hematopoiesis (CH). Importantly, ctDNA interpretation is subject to additional biological limitations including tumor shedding variability by anatomical site and disease burden, the timing of sample collection relative to surgery or chemotherapy, platform-specific detection thresholds, and pre-analytical variables such as hemolysis and delayed plasma processing. Randomized evidence supports ctDNA-guided de-escalation of adjuvant therapy in stage II colon cancer, with 5-year follow-up from the DYNAMIC trial demonstrating comparable recurrence-free survival (88% vs. 87%) and overall survival (93.8% vs. 93.3%) despite reduced chemotherapy use. In stage III disease, ctDNA is strongly prognostic, but chemotherapy intensification based solely on molecular positivity has not improved outcomes. This review additionally addresses underexplored areas including ctDNA in rectal cancer and neoadjuvant therapy, the influence of microsatellite instability (MSI) status on assay performance, and disparities in ctDNA access across racial, socioeconomic, and geographic populations.

**Conclusions:**

For gastroenterologists encountering ctDNA results in postoperative and surveillance settings, three principles should guide practice (i): match assay selection to clinical intent - tumor-informed platforms for MRD-directed adjuvant decisions, tumor-agnostic platforms when tissue is unavailable (ii); interpret results within biological and technical context, accounting for platform-specific sensitivity limitations, biological confounders, and quantitative VAF trajectory rather than binary thresholds; and (iii) integrate ctDNA findings into multidisciplinary pathways - de-escalation is supported by high-quality randomized evidence in stage II disease (Grade A; Level I), while chemotherapy escalation based solely on ctDNA positivity in stage III disease is not supported outside clinical trials (Grade C; Level I). Standardization of assay performance benchmarks and equitable access to testing remain critical unmet needs.

## 1 Introduction

Colorectal cancer (CRC) is the third most commonly diagnosed cancer and the second leading cause of cancer-related death worldwide, with an estimated 1.9 million new cases annually, based on GLOBOCAN 2022 data (1). Despite advances in surgical technique, adjuvant chemotherapy, and surveillance imaging, up to 30% of patients with stage II and 50% with stage III disease experience recurrence after curative-intent resection (2). This residual risk reflects the persistent challenge of detecting and responding to microscopic disease that is not captured by conventional staging tools.

The growing clinical availability of ctDNA assays has introduced a genuinely novel dimension to this problem. Circulating tumor DNA (ctDNA) is a tumor-derived subfraction of cell-free DNA (cfDNA) in plasma that carries the somatic mutational, copy number, and epigenomic signatures of the originating tumor cells (3). When detected after curative-intent surgery, it provides molecular evidence of persistent disease activity at a time when imaging, carcinoembryonic antigen (CEA) and clinical examination may all appear normal (1). This capability has generated considerable enthusiasm, but it has also created new clinical dilemmas: not all positive tests mean the same thing, not all negative tests provide the same reassurance, and the appropriate response to a ctDNA result depends critically on which assay was used, when it was drawn, and what clinical question it was designed to answer (2).

The two principal assay categories, tumor-informed and tumor-agnostic, differ substantially in analytical approach, performance characteristics, tissue requirements, and turnaround times (2). A recent systematic review and diagnostic accuracy meta-analysis found that in serial-sampling settings, tumor-informed assays achieve significantly higher pooled sensitivity than tumor-agnostic approaches (88% vs. 59%, p=0.001) (4); the authors note substantial heterogeneity across the pooled studies in platform design, patient populations, and follow-up protocols, underscoring that platform choice is not a trivial decision and that these figures should be interpreted as aggregate estimates rather than fixed performance guarantees. At the same time, landmark randomized trials including DYNAMIC and DYNAMIC-III have begun to define where ctDNA testing changes clinical management and where it does not, providing a necessary evidence-based framework for interpreting individual results (5–7).

Despite this progress, several clinically important areas remain incompletely addressed in existing reviews, including ctDNA application in the rectal cancer and neoadjuvant therapy context, the distinct interpretive challenges introduced by microsatellite instability (MSI) status, and the disparities in ctDNA access that limit equitable implementation across diverse patient populations (8, 9). Gastroenterologists are increasingly encountering ctDNA results in clinical practice, often in postoperative and surveillance settings, without standardized institutional guidance on test selection or result interpretation.

This review examines the biological basis of ctDNA shedding, the technical landscape of available assay platforms, the stage-specific clinical evidence base, practical implementation considerations, and evidence gaps that remain unresolved. The goal is to equip gastroenterologists with a structured, evidence-grounded framework for incorporating ctDNA into post-treatment CRC management in 2026.

## 2 Methods

### 2.1 Search strategy

We conducted a comprehensive narrative review of the literature using PubMed, Embase, and the Cochrane Library from January 2015 through March 2026. Search terms were applied using Boolean operators and included: “circulating tumor DNA,” “ctDNA,” “cell-free DNA,” “liquid biopsy,” “minimal residual disease,” “colorectal cancer,” “colon cancer,” “rectal cancer,” “tumor-informed assay,” “tumor-agnostic assay,” “clonal hematopoiesis,” “microsatellite instability,” and “MRD.” We also searched ClinicalTrials.gov for ongoing and recently completed trials using ctDNA endpoints in CRC. Reference lists of included articles were reviewed to identify additional relevant sources.

While this manuscript is a narrative rather than a systematic review, the search strategy was conducted using predefined keyword combinations applied to each database independently. Two authors (A.S. and S.M.) independently screened titles and abstracts against the inclusion criteria defined in Section 2.2, with disagreements resolved by discussion. The search terms, screening decisions, and final reference list are available from the corresponding author upon request. This approach was designed to minimize selection bias while retaining the narrative synthesis format appropriate for the clinical integration goals of this review.

### 2.2 Study selection

We included (1): randomized controlled trials evaluating ctDNA-guided therapy selection or de-escalation (2); prospective cohort studies of ctDNA performance characteristics in localized or metastatic CRC (3); systematic reviews and meta-analyses reporting ctDNA sensitivity, specificity, or predictive value (4); clinical practice guidelines from major oncology societies; and (5) foundational studies establishing the biological basis of ctDNA shedding and detection. We excluded case reports, studies enrolling fewer than 20 patients, and studies not reporting clinically applicable ctDNA outcomes. Non-peer-reviewed sources, conference abstracts without subsequent full publication, and preprints are identified as such where cited. When studies reported conflicting findings, we prioritized evidence from randomized controlled trials and larger prospective cohorts and explicitly noted areas of uncertainty in the narrative synthesis.

### 2.3 Data synthesis

Given the heterogeneity of study designs, assay platforms, and clinical applications in the included literature, we performed a narrative synthesis organized by assay type and clinical stage of disease. Evidence quality is noted for key recommendations using a three-level grading framework: Level I (randomized controlled trials), Level II (prospective cohort studies), and Level III (retrospective studies and expert consensus). This review adheres to the general principles of good narrative review practice and references REMARK criteria for prognostic biomarker reporting where applicable (10).

## 3 Biological basis of ctDNA shedding in colorectal cancer

Circulating tumor DNA (ctDNA) is a subfraction of the total cfDNA found in plasma and other bodily fluids that originates specifically from tumor cells. Tumor cell death through apoptosis and necrosis is the predominant driver of ctDNA release into the bloodstream, though non-apoptotic secretion pathways have also been described (3). Once in circulation, tumor-derived DNA fragments are cleared rapidly, with half-lives estimated at under 20 minutes to roughly 2.5 hours (11). This fast turnover is a defining biological feature: ctDNA concentrations shift quickly in response to tumor dynamics, making it a marker of current disease burden rather than a fixed historical record.

Tumor shedding is not uniform. Several factors govern how much ctDNA enters the bloodstream, including tumor size, stage, anatomical location, vascularization, and the degree of necrosis (3). In metastatic CRC, ctDNA may comprise a meaningful fraction of total cfDNA, sometimes several percent by variant allele frequency (VAF). In early-stage or localized disease, however, VAF may fall well below 0.01%, demanding assays with extremely high analytical sensitivity (3, 11). This has direct implications for test selection: assays capable of detecting very low VAF signals are essential in the postoperative minimal residual disease (MRD) setting, where ctDNA levels can be exceedingly low even when microscopic residual disease is biologically active.

Tumor location also influences shedding dynamics in ways that are clinically relevant. The liver receives portal venous drainage from the colon, which may explain in part why liver metastases from CRC tend to shed more ctDNA into the circulation than lung or peritoneal metastases. This anatomical gradient accounts for the site-specific sensitivity differences observed in plasma-only assays, where liver recurrence is detected at close to 100% sensitivity compared to approximately 40% for peritoneal disease (12). Understanding this biological variability helps gastroenterologists interpret negative results more critically when the clinical context suggests a higher risk of extra-hepatic or peritoneal recurrence.

Fragment length analysis adds another layer of biological complexity. Tumor-derived cfDNA shows a distinct fragmentation pattern compared to normal cfDNA released from non-malignant cells, a feature being leveraged by emerging machine learning tools to improve ctDNA detection at low VAF without relying solely on mutational content (13). These fragmentomics-based approaches are still predominantly in the research phase, but they illustrate how the biological properties of ctDNA continue to generate new analytical strategies with genuine clinical potential. Further details on the technical platforms that exploit these features are described in Section 6.

## 4 Where ctDNA fits in CRC care in 2026: the evidence base has matured

ctDNA progressed from a prospective research tool to a valid clinical biomarker as sequencing sensitivity increased and prospective longitudinal studies revealed a robust relationship between postoperative ctDNA detection and recurrence risk (1). The principal clinical studies informing current ctDNA use in CRC, and the corresponding levels of evidence, are summarized in **Supplementary Table 1**.

Randomized results support a ctDNA-guided approach to reduce unnecessary treatment for stage II colon cancer. The DYNAMIC trial found that ctDNA-guided care decreased adjuvant chemotherapy use from 28% to 15% without affecting 2-year recurrence-free survival rates (93.5% vs. 92.4%) (5). This was a well-designed randomized controlled trial with prospective ctDNA testing, blinded allocation to treatment groups, and intention-to-treat analysis, conferring low risk of bias (Level I evidence). Longer follow-up has reinforced confidence in this approach. At five years, recurrence-free survival (88% vs. 87%) and overall survival (93.8% vs. 93.3%) remained indistinguishable between the two management strategies (6). Among ctDNA-positive patients who received chemotherapy, molecular clearance was documented in 87.5% by the time therapy ended, and those in whom ctDNA remained detectable at that point formed a distinctly higher-risk subgroup (6).

In stage III colon cancer, ctDNA has gained broad acceptance as a strong prognostic tool, though its therapeutic utility remains more difficult to define. In DYNAMIC-III, a single plasma draw at 5–6 weeks after surgery stratified patients into two groups with strikingly divergent 3-year recurrence-free survival: 87% in the ctDNA-negative group versus 49% in those with detectable ctDNA (7). Two key therapeutic conclusions emerged. First, intensifying chemotherapy in ctDNA-positive patients produced no survival advantage over standard management (2-year recurrence-free survival 51% vs. 61%). Second, de-escalating oxaliplatin-based therapy in ctDNA-negative patients reduced drug exposure (34.8% vs. 88.6% receiving oxaliplatin) and hospitalizations (8.5% vs. 13.2%), but the de-escalation arm did not satisfy its prespecified noninferiority threshold (7). Among ctDNA-positive patients, those with persistent molecular positivity after completing therapy fared considerably worse (3-year recurrence-free survival 14% vs. 79%) (7). This was a prospective randomized phase 2/3 trial with low overall risk of bias; its adaptive design limits some direct comparisons, and control arm modification should be acknowledged when interpreting results (Level I evidence for prognostic value; Level II for therapeutic guidance).

Extensive prospective observational cohorts continue to support these findings. A prospective, multicenter study of stage II/III CRC using serial sampling found ctDNA positivity to be the most accurate independent predictor of recurrence, with high hazard ratios at multiple time points. Serial ctDNA indicated recurrence before radiologic imaging, with a mean lead time of 5.01 months (14). While this prospective cohort demonstrated strong prognostic value, the absence of randomization limits causal inference (Level II evidence).

For gastroenterologists, the implication is clear: ctDNA has moved beyond an experimental biomarker. A positive result during disease-eradication follow-up often indicates biologically active disease even when imaging is normal. The most challenging step is selecting the appropriate assay and interpreting results in the context of the clinical question.

## 5 Why assay selection matters: “positive” and “negative” are not universal

Selecting the assay is more than a technical concern. It defines what “positive” and “negative” mean for a specific patient and clinical setting (2).

Result interpretation must account for both false-positive and false-negative risks, which differ by clinical context. In the postoperative MRD setting, where ctDNA concentrations are often exceedingly low, the predominant analytical challenge is the false-negative: failure to detect residual disease at sub-threshold VAFs. Conversely, false-positive signals can arise from multiple non-tumor sources and carry real consequences for patient anxiety, unnecessary imaging, invasive procedures, and premature therapy escalation (2).

Biological and physiological confounders of ctDNA include: (1) pre-analytical variables - hemolysis from suboptimal blood handling, leukocyte lysis releasing germline DNA, and delayed plasma separation (>2 hours), all of which dilute or contaminate the cfDNA fraction; (2) physiological elevations - vigorous physical exercise, surgical trauma, systemic inflammation, and neuroendocrine tumors (NETs) can transiently increase cfDNA from non-tumor sources; (3) host-related factors - advancing age, higher BMI, male sex, and hepatic dysfunction alter cfDNA clearance kinetics and baseline concentrations, potentially affecting assay thresholds; and (4) biological confounders such as clonal hematopoiesis (CH), detailed further in Section 11.

An important conceptual shift is underway in ctDNA interpretation: the field is progressively moving beyond a binary positive/negative framework toward quantitative variant allele frequency (VAF) and ctDNA concentration monitoring. Emerging data demonstrate that the absolute ctDNA concentration and VAF trajectory - rather than simple detection alone - correlate with distinct survival outcomes. In prospective analyses, patients with higher baseline ctDNA concentrations at MRD timepoints exhibit significantly worse recurrence-free survival than those with low-positive results, and rising VAF trajectories between serial draws confer markedly inferior outcomes compared to declining or stable signals. This quantitative dimension has direct implications for result interpretation: a single low-level positive at a VAF of 0.01–0.05% should not prompt the same clinical response as a persistently rising signal at 0.5% VAF. Gastroenterologists should request quantitative VAF data from laboratory reports when available and frame serial results as a trajectory rather than a binary event.

Clonal hematopoiesis (CH) is a vital biological confounder that can generate false positives. Age-related clonal expansions of hematopoietic cells may leak mutant DNA into the plasma, leading to alterations that, if not properly filtered, might be misidentified as tumor-derived ctDNA. This process has a considerable impact on test design and result interpretation, requiring matched white blood cell (WBC) sequencing and strict variant filtering (15). Studies show that up to 42% of cancer patients harbor CH variants, with certain DNA repair genes including BRCA2 (39% of variants), CHEK2 (38%), and ATM (20%) frequently affected, potentially leading to false-positive results and inappropriate treatment recommendations if not properly filtered (16, 17).

The kind of test used may also alter the apparent lead time for recurrence detection. ctDNA may detect relapses earlier than imaging in some settings; however, this advantage may be diminished when imaging closely follows established surveillance schedules. A retrospective analysis of surveillance strategies found that ctDNA did not significantly outperform imaging combined with CEA when monitoring followed established schedules, indicating that real-world effectiveness is partly dependent on what is already included in the surveillance program (18).

Experience with interventional research has also shown that test performance and population selection can significantly affect clinical outcomes. The phase II analysis of the NRG-GI005 (COBRA) trial for resected stage IIA colon cancer did not meet its prespecified endpoint, leading to enrollment discontinuation per stopping criteria with the original assay, underscoring the importance of assay specificity in low-risk patient populations (19).

## 6 Understanding assay types: tumor-informed versus tumor-agnostic

ctDNA assays used for MRD assessment and surveillance fall into two broad approaches, and the best choice depends on the clinical question and practical constraints (2). **Table 1** provides a comparative summary of the principal assay platforms, including commercially available examples.

**Table 1:** Comparative overview of ctDNA assay approaches for MRD detection and surveillance in CRC.

Feature	Tumor-informed	Tumor-agnostic (fixed panel NGS)	Tumor-agnostic (epigenomic/methylation)
Commercial examples	Signatera (Natera); FoundationOne Tracker (Foundation Medicine, up to 50-variant panel)	Guardant360 CDx (Guardant Health); Resolution Bioscience ctDx platforms	Guardant Reveal (Guardant Health); Shield (Grail)
Tissue requirement	Yes; archival tumor for personalized panel design	No	No
Assay design turnaround	2–4 weeks (tumor sequencing + personalized panel synthesis)	Within 1–2 weeks	Within 1–2 weeks
Analytical sensitivity	Very high; detects VAF <0.01% with matched WBC filtering	High; may miss private mutations not on panel	High for common recurrence patterns; site-dependent
Serial sampling sensitivity (meta-analysis)	88%	59%	81% longitudinal sensitivity (12)
Specificity	Very high; private mutations substantially reduce false positives	High; CH filtering quality varies by platform	Very high; 98.2% in prospective evaluation (12)
Site-specific sensitivity	Broadly consistent across anatomical recurrence sites	Variable; depends on mutations covered by fixed panel	Liver 100%, lung 53%, peritoneal 40% (12)
CH filtering	Inherent through matched WBC sequencing	Varies by platform; may require separate bioinformatic algorithm	Epigenomic signals inherently less vulnerable to CH interference
Best clinical scenario	MRD detection post curative-intent surgery; high-specificity surveillance	Rapid access needed; tissue unavailable; initial assessment	No tissue available; early surveillance; methylation-preferred setting
Key limitation	Requires tissue access; longer time to first result	Lower sensitivity for private mutations; inconsistent CH filtering	Site-dependent sensitivity; less mature long-term outcome data
Approximate cost (USD)	$3,000–$5,000 (NGS-based); ddPCR-based panels: $500–$1,500 (Section 10.3)	$1,500–$3,500	$1,500–$3,000
Key references	(2, 4, 21)	(2, 4)	(12, 17)

CH, clonal hematopoiesis; MRD, minimal residual disease; NGS, next-generation sequencing; VAF, variant allele frequency; WBC, white blood cell. Commercial platforms listed are examples only and do not constitute endorsement; characteristics reflect manufacturer specifications and published validation studies as of March 2026. Pricing is approximate and subject to change.

### 6.1 Tumor-informed assays: high specificity when MRD is the question

In a tumor-informed workflow, resected tumor tissue undergoes sequencing to define a set of patient-specific somatic variants, which are then tracked longitudinally across serial plasma samples. Because the monitored variants are private to that individual’s tumor, background noise from non-tumor-derived cfDNA is substantially reduced, giving this approach a major advantage in specificity. This matters most in the postoperative MRD context, where ctDNA concentrations are often very low and a false-positive result carries real clinical and psychological costs (2).

Commercially available tumor-informed platforms include Signatera (Natera), which uses whole-exome sequencing of tumor tissue to design a personalized 16-variant panel tracking clonal somatic mutations, and FoundationOne Tracker (Foundation Medicine), which similarly uses tumor sequencing to build a personalized panel of up to 50 somatic variants selected from whole-exome data. Both platforms employ matched WBC sequencing to filter clonal hematopoiesis variants, though the number of tracked variants and bioinformatic filtering algorithms differ between them.

The logistical tradeoff is significant. Tumor-informed testing requires available tissue, tumor sequencing, and assay design before obtaining the first plasma result, a lead time of two to four weeks that may be clinically significant during time-sensitive adjuvant decision windows (2).

### 6.2 Tumor-agnostic assays: speed and accessibility, with performance that can vary by site

Unlike their tumor-informed counterparts, tumor-agnostic platforms operate entirely from plasma, with no archival tissue required. Older designs rely on panels covering a fixed set of recurrently mutated cancer genes; more recently developed tissue-free assays layer in epigenomic and methylation-based signals, sometimes alongside genomic mutation data, to capture a broader molecular signal from the tumor (12). The practical benefit is speed and wider accessibility, particularly when no tissue was collected prospectively or when a rapid result is needed. Commercially available platforms in this category include Guardant Reveal (Guardant Health), which combines methylation profiling with targeted mutation detection, and Shield (Grail), which uses a methylation-based approach originally developed for multi-cancer detection.

The limitation is that performance can vary by recurrence biology and anatomical site. In an extensive evaluation of a tissue-free epigenomic MRD assay using longitudinal postoperative samples, longitudinal sensitivity in stage II or higher colon cancer was 81%, the median lead time was 5.3 months, and sensitivity differed markedly by recurrence site (liver 100%, lung 53%, peritoneal 40%), while specificity remained high (98.2%) (12). These differences matter when deciding how much reassurance a negative result should provide, particularly in patients at risk for lung-dominant or peritoneal recurrence.

### 6.3 Technical approaches: how ctDNA assays work

Three principal methodological frameworks underpin current assay platforms (13, 20).

Digital droplet polymerase chain reaction (ddPCR) partitions a DNA sample into thousands of individual droplets and amplifies each independently, enabling absolute quantification of rare mutant alleles at VAFs as low as 0.01% (21). This approach has been used to track known driver mutations such as KRAS, NRAS, and BRAF variants with high reproducibility (21). Its inherent limitation is that it is targeted: only pre-selected mutations can be tracked, making it unsuitable for patients whose tumors carry rare private mutations not included in the panel.

Next-generation sequencing (NGS) approaches range from targeted gene panels covering tens to hundreds of cancer-associated genes to whole-exome designs. Tumor-informed NGS platforms derive a patient-specific mutation panel from prior tissue sequencing and then use targeted deep sequencing of plasma DNA to track those exact variants, achieving very low false-positive rates through dual filtering against matched WBC DNA (2). Tumor-agnostic panel-based NGS assays use fixed mutation sets without requiring tissue, enabling faster turnaround at the cost of lower sensitivity in patients whose tumors carry private mutations not covered by the panel (2, 22).

Methylation and epigenomic profiling exploit the distinct epigenomic signatures of cancer cells, including aberrant CpG methylation at gene promoters, to identify tumor-derived cfDNA without requiring prior knowledge of tumor-specific mutations. These assays are fully tissue-free and can be applied even when archival tissue is unavailable (12). A prospective evaluation in CRC demonstrated 81% longitudinal sensitivity with 98.2% specificity and a 5.3-month median imaging lead time (12), though site-specific sensitivity limitations remain a key consideration.

**Figure 1:**
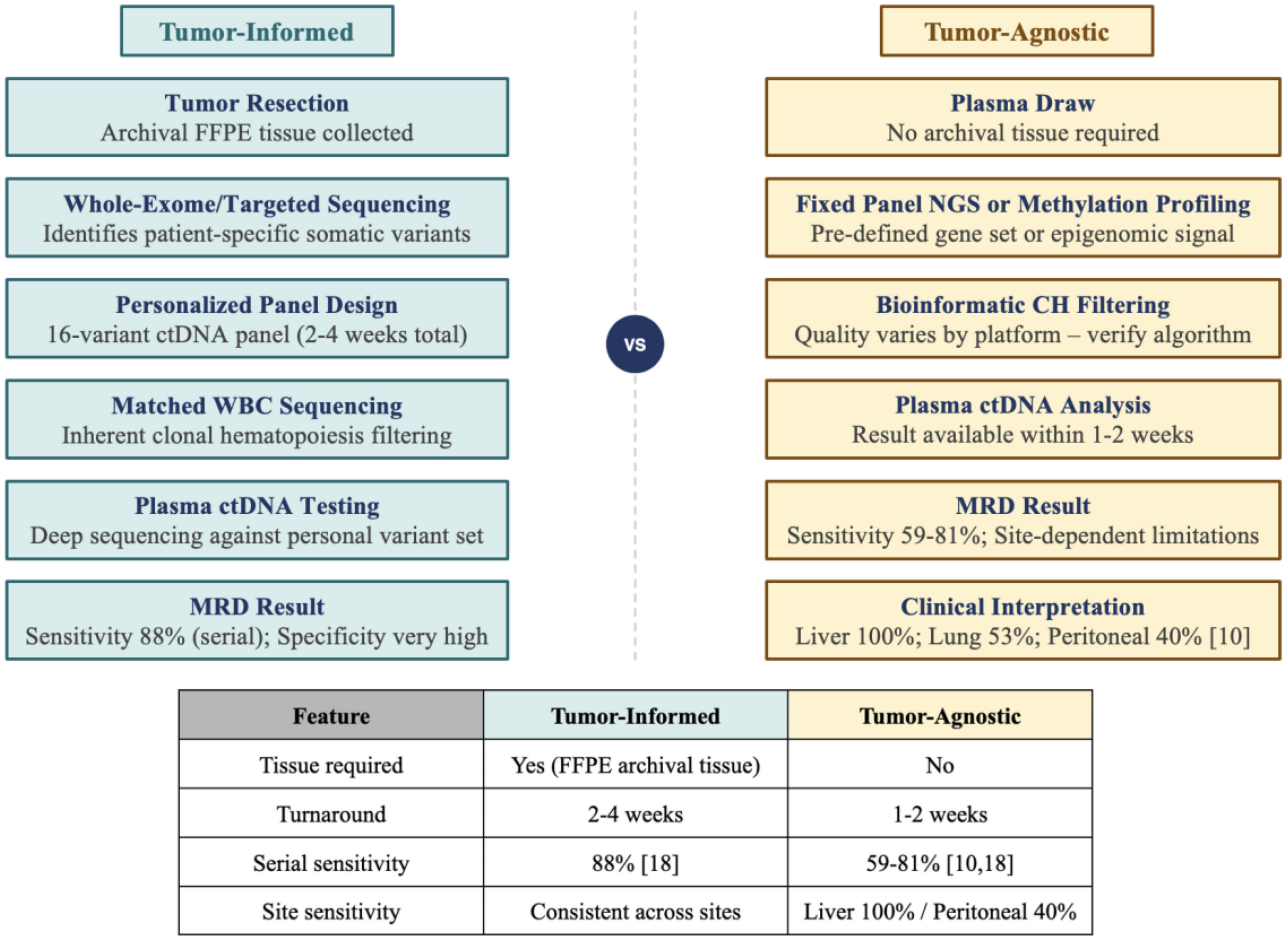
Comparative workflow and performance characteristics of tumor-informed versus tumor-agnostic ctDNA assays. A two-column side-by-side workflow diagram comparing tumor-informed and tumor-agnostic ctDNA assay processes. Left column (Tumor-Informed): Tumor resection → Archival FFPE tissue → Whole-exome or targeted tumor sequencing (2–4 weeks) → Patient-specific variant panel design → Plasma ctDNA testing with matched WBC filtering → MRD result. Right column (Tumor-Agnostic): Plasma draw → Fixed mutation panel or methylation/epigenomic profiling → CH bioinformatic filtering (quality varies by platform) → MRD result. Key performance metrics from **Table 1** are annotated on each pathway. Tumor-informed assays require archival tissue and personalized panel design but offer higher specificity for MRD detection (sensitivity 88%). Tumor-agnostic assays are tissue-free and faster but may have site-specific sensitivity limitations (peritoneal recurrence ~40%) (2, 4, 12). CH, clonal hematopoiesis; ctDNA, circulating tumor DNA; FFPE, Formalin-Fixed Paraffin-Embedded; MRD, minimal residual disease; NGS, Next-Generation Sequencing; WBC, white blood cell.

Hybrid platforms combining tumor-informed mutation tracking with epigenomic signals are under active analytical validation, with early data suggesting improved sensitivity over either strategy alone (17). These hybrid approaches may represent the next generation of clinical assays, though prospective outcome data are not yet available (**Figure 1**).

## 7 Stage-specific clinical use: matching the test to the decision

### 7.1 Stage II colon cancer: reducing overtreatment with randomized support

Randomized data firmly support ctDNA-guided treatment decisions, with the primary therapeutic goal of preventing unnecessary chemotherapy. DYNAMIC demonstrated that patients could receive less chemotherapy while retaining comparable cancer-free survival at two years (5). The five-year results confirmed the same for long-term recurrence-free and overall survival (6) (Grade A recommendation; Level I evidence). In this setting, the test must perform reliably within the MRD detection range and deliver results quickly enough to inform adjuvant selection before the treatment window closes.

Per current NCCN Guidelines (v2.2026), this de-escalation pathway represents the only setting in which a specific therapeutic action based on ctDNA results is supported by Level I randomized evidence; outside this context, ctDNA results should inform risk stratification and surveillance intensity rather than direct unilateral treatment decisions (see Sections 7.2–7.4).

### 7.2 Stage III colon cancer: strong prognostication, but escalation is not a fix

In stage III disease, ctDNA is strongly prognostic after surgery and after chemotherapy. A correlative study showed that postoperative ctDNA positivity and post-chemotherapy ctDNA positivity were both associated with substantially worse recurrence outcomes (23). DYNAMIC-III further validated ctDNA as a high-impact risk classifier: ctDNA-positive patients had threefold-higher recurrence rates, and simply intensifying conventional chemotherapy based on ctDNA positivity did not improve recurrence-free survival compared with standard management (7) (Grade B recommendation for prognostic use; Grade C for therapeutic escalation; Level I evidence).

For gastroenterologists, this is where counseling matters. A positive ctDNA test signals high risk, but it does not automatically dictate a single proven intervention outside a clinical trial. Consistent with NCCN v2.2026 guidance, the appropriate response to a positive ctDNA result is early oncology coordination, confirmation of clonal hematopoiesis filtering, a repeat ctDNA draw in 2–4 weeks to assess trajectory [since persistence or a rising VAF is far more clinically actionable than a single low-level positive that subsequently resolves (14)], and surveillance planning that is appropriately intensified rather than reflexive. Patients with persistent ctDNA positivity across multiple measurements may be candidates for early referral to clinical trials; intensification of standard adjuvant chemotherapy based solely on ctDNA positivity is not supported and should not be applied outside of clinical trials (7).

### 7.3 Surveillance after curative-intent therapy: trends are more reliable than single values

For surveillance, the most reliable information comes from trajectory, not a single result. Serial ctDNA testing outperforms isolated measurements because persistent positivity or rising levels are far more clinically meaningful than a one-off low-level positive that subsequently disappears (14). In the multicenter observational cohort noted above, serial ctDNA achieved high overall accuracy for recurrence detection and provided a mean lead time of approximately 5 months ahead of imaging (14) (Level II evidence).

Assay choice during surveillance should also reflect anticipated recurrence patterns. In settings where lung-dominant or peritoneal recurrence is plausible, site-dependent sensitivity limitations seen in some plasma-only assays mean a negative ctDNA result cannot replace guideline-directed imaging (12).

ctDNA should be viewed by gastroenterologists as a biological risk indicator rather than a radiological diagnostic tool. A positive ctDNA test reveals molecular evidence of disease activity but does not automatically correspond with clinically visible recurrence; a negative result does not exclude recurrence, particularly in cases involving low-volume disease or recurrence patterns less sensitive to plasma shedding. When a positive ctDNA result is encountered without corresponding imaging findings, a structured approach is warranted: confirm the assay timing relative to surgery or chemotherapy (since different clinical objectives correspond to different landmark sampling periods), clarify whether the assay was tumor-informed or tumor-agnostic and whether CH filtering was applied, and retest ctDNA to determine whether the result persists or changes (12, 14, 15, 18).

### 7.4 Metastatic and oligometastatic settings: promise is real, pathways are still evolving

ctDNA has established a growing role in metastatic CRC for therapy selection, resistance monitoring, and prognostication. One of its most clinically actionable applications in this setting is the detection of emergent resistance mutations during anti-EGFR therapy. Acquired RAS and BRAF mutations, which confer resistance to cetuximab and panitumumab, can be detected in plasma ctDNA weeks to months before radiographic progression, enabling earlier therapeutic switching and potentially prolonging disease control (20, 24). Serial ctDNA monitoring during systemic therapy can also serve as a pharmacodynamic marker, with early ctDNA clearance correlating with radiographic response and improved survival in several prospective analyses (25).

Standardized gastroenterology-driven pathways for ctDNA use in the metastatic setting are less mature than in the nonmetastatic MRD context. Contemporary reviews emphasize that, while the prognostic signal is strong, how best to act on ctDNA results in metastatic disease should remain anchored in multidisciplinary decision-making (26, 27).

### 7.5 Rectal cancer and neoadjuvant therapy settings: a distinct clinical landscape

Most published ctDNA studies in CRC have enrolled mixed colon and rectal cancer populations or have focused predominantly on colon cancer. Rectal cancer presents a distinct clinical context that shapes both when and how ctDNA should be applied (28).

The use of total neoadjuvant therapy (TNT) in locally advanced rectal cancer, combining long-course chemoradiation with induction or consolidation chemotherapy before surgery, has become increasingly standard following the OPRA trial, which demonstrated higher rates of organ preservation with TNT versus chemoradiation alone (28). This treatment sequence creates a meaningful opportunity to evaluate ctDNA as a pharmacodynamic and predictive marker. The central clinical question is whether ctDNA clearance during or after TNT can reliably predict pathological complete response (pCR) and thereby inform organ-preservation decisions.

The watch-and-wait strategy for patients who achieve a clinical complete response after TNT has been validated in cohort studies, but distinguishing true pCR from near-complete response using imaging and endoscopy alone remains a significant challenge (28). Preliminary data suggest that ctDNA clearance after TNT is associated with pCR and durable local regrowth-free survival, though sensitivity at this time point can be limited by low tumor burden (29). The ongoing DYNAMIC-Rectal trial and IMPROVE-IT2 trial (NCT04543604) are prospectively evaluating ctDNA-guided decision-making in this setting, with results anticipated to clarify the role of molecular clearance in supporting organ-preservation decisions.

For gastroenterologists who co-manage rectal cancer patients considering non-operative management, understanding the current possibilities and limitations of ctDNA is increasingly relevant. Postoperatively, rectal cancer patients require endoscopic surveillance in addition to imaging, and ctDNA adds a molecular layer to this program. The principles governing assay selection, timing, and interpretation discussed throughout this review apply broadly, though rectal-specific prospective data remain less mature than in colon cancer.

## 8 Microsatellite instability and its effect on ctDNA testing

Approximately 15% of CRCs are MSI-high (MSI-H) or mismatch repair deficient (dMMR), a characteristic with major implications for prognosis, treatment selection, and ctDNA test performance (30). MSI-H tumors harbor a markedly elevated somatic mutation burden due to defective DNA mismatch repair. In principle, this provides a richer mutational target landscape for tumor-informed ctDNA assays, since there are more patient-specific variants available for personalized panel design.

In practice, however, several important considerations apply. First, stage II MSI-H colon cancers have a favorable prognosis, and current guidelines recommend against routine adjuvant chemotherapy in this group. The ctDNA-guided de-escalation framework demonstrated in DYNAMIC was applied to a predominantly microsatellite stable (MSS) population, and whether the same strategy translates equally to MSI-H stage II disease requires dedicated analysis (5). Applying ctDNA-guided adjuvant decision-making to MSI-H stage II patients without specific evidence risks misclassifying a patient who has a naturally favorable prognosis as one requiring treatment escalation.

Second, immunotherapy with immune checkpoint inhibitors (ICIs) has transformed the management of dMMR/MSI-H metastatic CRC. In the KEYNOTE-177 trial, pembrolizumab demonstrated superior progression-free survival over standard chemotherapy as first-line therapy for MSI-H/dMMR metastatic CRC (30). In this setting, ctDNA has shown early promise as a pharmacodynamic marker of ICI response, with ctDNA clearance correlating with radiographic response and improved survival outcomes (30, 31). Serial ctDNA monitoring during ICI therapy may provide earlier signal of response or progression than imaging alone, enabling more timely therapeutic decisions, though this application is not yet standardized for routine practice outside clinical trials.

Third, ctDNA shedding patterns may differ by mismatch repair status. Some data suggest that MSI-H tumors can shed ctDNA at different rates or VAFs compared to MSS tumors of similar stage and size, which could affect assay performance at standard analytical thresholds (31). Gastroenterologists should be aware that MMR status, routinely assessed by immunohistochemistry or PCR as part of standard CRC pathology evaluation, is a relevant factor when interpreting ctDNA results and when discussing those results with patients and oncology colleagues.

Emerging computational tools, including deep learning models trained on histopathological features, have demonstrated the ability to predict MSI status and tumor mutational burden from digitized tissue images in digestive tract cancers, offering a complementary molecular characterization approach that may be particularly useful when standard IHC- or PCR-based MMR testing is unavailable or inconclusive. Integration of such AI-based MSI classifiers with ctDNA platforms represents a promising direction for comprehensive molecular profiling in CRC management (32).

## 9 Health equity and access disparities in ctDNA testing

The clinical promise of ctDNA cannot be realized uniformly if access to testing is unequal. Existing data suggest that disparities in liquid biopsy utilization follow well-documented patterns of inequity in oncology care, with access gaps by race, ethnicity, insurance status, geographic region, and socioeconomic background (8, 9).

Coverage policies remain inconsistent. A 2023 analysis found that while most private payer and Medicare policies covered at least one ctDNA indication, coverage for MRD-specific testing was provided in only 65% of those policies addressing this indication and was frequently restricted to scenarios where tissue biopsy was contraindicated or unavailable (33). Patients with Medicaid coverage, who are disproportionately low-income and more likely to be from racial and ethnic minority backgrounds, may face additional barriers not reflected in commercial trial cohorts. The cost per ctDNA test ranges from approximately $1,500 to $5,000 depending on platform and indication, making unreimbursed testing cost-prohibitive for many patients (27). These gaps can result in testing delays, unanticipated out-of-pocket expenses, and real-world disparities in who benefits from emerging molecular surveillance tools.

Geographic disparities compound these access challenges. Academic medical centers with established liquid biopsy pathways and multidisciplinary tumor boards are concentrated in urban settings. Rural and community-based gastroenterology and oncology practices may lack the infrastructure, reference laboratory relationships, or institutional familiarity with assay workflows needed to incorporate ctDNA testing consistently (9). This creates a two-tier system in which access to molecular surveillance tools depends heavily on where a patient receives care.

Trial enrollment data also raise important concerns. The landmark ctDNA studies including DYNAMIC, DYNAMIC-III, COBRA, and major observational cohorts were conducted predominantly in high-resource academic settings in Australia, Europe, and the United States, with limited representation of Black, Hispanic, and Asian patients (9). Since tumor mutational profiles and ctDNA shedding characteristics may differ by ancestry, the generalizability of current performance benchmarks to diverse populations has not been fully established.

Several patient assistance programs are available to help mitigate out-of-pocket costs. Natera offers a patient assistance program for Signatera, and Guardant Health provides financial counseling for Guardant Reveal. Care teams should proactively assess insurance coverage, understand prior authorization requirements, and connect patients with these resources when applicable. At an institutional level, advocating for universal coverage policies, establishing partnerships with community oncology practices, and integrating ctDNA ordering into a shared decision-making framework that accounts for cost and access are practical steps toward more equitable implementation.

## 10 Practical implementation in 2026: timing, tissue, and workflow

### 10.1 When to draw: timing shapes interpretability

Timing influences interpretability because postoperative and post-treatment cfDNA dynamics can affect the signal-to-noise ratio. Many practice-defining studies anchor MRD testing in the early postoperative window. DYNAMIC used week 4 and week 7 samples after surgery, with treatment decisions based on those results (5). DYNAMIC-III sampled at 5–6 weeks after surgery (7). Practice pathways commonly aim for a similar 4-8-week window when the goal is MRD-directed adjuvant decision-making (5, 7, 12).

**Figure 2:**
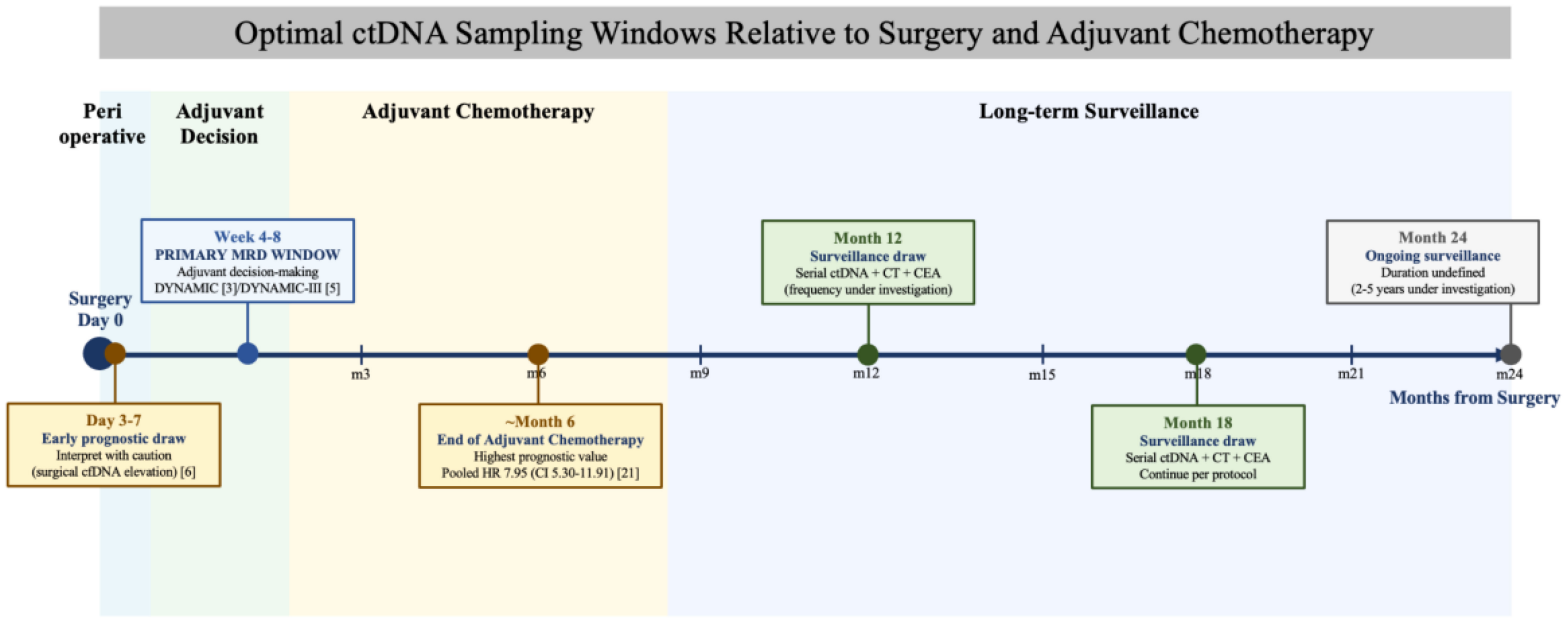
Optimal ctDNA sampling windows relative to surgery and adjuvant chemotherapy. A horizontal timeline from the day of surgical resection through 24 months of follow-up illustrating evidence-based ctDNA sampling windows. Key anchor points include: Day 3-7 [early prognostic draw; interpret with caution given potential surgical cfDNA elevation (14)]; Week 4–8 post-surgery [primary MRD window for adjuvant decision-making (5, 7)]; End of adjuvant chemotherapy [highest prognostic value; pooled HR 7.95, 95% CI 5.30-11.91 for identifying high-risk patients (34)]; 6-monthly intervals thereafter (surveillance phase). Time points are based on landmark trials [DYNAMIC (5), DYNAMIC-III (7)] and prospective cohort studies (14). Serial surveillance sampling intervals beyond the post-adjuvant window remain under investigation. CEA, Carcinoembryonic Antigen; cfDNA, cell-free DNA; CT, Computed Tomography; ctDNA, circulating tumor DNA; MRD, minimal residual disease; HR, hazard ratio.

Very early postoperative testing can also be prognostic. In an extensive multicenter observational study, ctDNA positivity at day 3–7 after surgery was associated with markedly increased recurrence risk (14). Those early results should be interpreted carefully within the clinical context, since surgical trauma may transiently elevate cfDNA from non-tumor sources, and assay limitations must be understood before acting on very early draws (**Figure 2**).

### 10.2 Tissue availability: a practical determinant of assay choice

When tumor tissue is readily available and the clinical question concerns MRD following curative-intent treatment, tumor-informed testing is often preferred due to its high specificity (2, 5). In the absence of tissue, or if the patient presents post-surgery without prior planning, a tumor-agnostic approach may be more practical, provided its performance limitations regarding recurrence biology and site-specific sensitivity are explicitly considered alongside imaging schedules (12, 23, 26).

### 10.3 Cost, insurance, and access: the assay is only helpful if patients can get it

Both analytical effectiveness and real-world applicability determine the clinical significance of ctDNA testing. Coverage policies vary significantly by location, healthcare system, and payer. Even as ctDNA testing for MRD becomes more widely discussed in clinical consensus recommendations, reimbursement for serial surveillance testing remains inconsistent, especially when stringent payer criteria are not met (35–37). A 2023 analysis found that coverage for MRD-specific testing was provided in only 65% of payer policies addressing this indication (33).

Operationally, tumor-informed assays require acquisition of archival tumor tissue, collaboration with pathology services, tumor sequencing, and personalized panel design before plasma testing begins (2, 5). Without institutional pathways, ordering practices can become inconsistent, leading to variable responses to positive results. Inclusion of ctDNA data at multidisciplinary tumor board discussions facilitates appropriate integration of molecular findings with imaging, pathology, and systemic treatment decisions, and helps reduce practice variability (38).

From a methodological standpoint, ddPCR and NGS differ substantially in cost structure and clinical evidence base. ddPCR assays for tracking known driver mutations (e.g., KRAS, NRAS, BRAF) are generally lower in per-test cost ($500–$1,500) and offer rapid turnaround, but are limited to pre-selected variants and cannot track private mutations absent from the panel. In contrast, targeted NGS panels and whole-exome–based tumor-informed assays are more expensive ($1,500–$5,000) but offer broader mutational coverage and the ability to detect novel resistance mutations. Critically, the randomized trial evidence base for ctDNA-guided management (DYNAMIC, DYNAMIC-III, COBRA) was generated exclusively using NGS-based tumor-informed or tumor-agnostic platforms; no randomized trials have utilized ddPCR as the primary ctDNA measurement technology for treatment decision-making. This means the actionable thresholds and clinical decision frameworks supported by current guidelines were derived from NGS data and should not be assumed to translate directly to ddPCR-based results without dedicated validation.

## 11 Clonal hematopoiesis: a critical source of false positives

CH is a more widespread source of false-positive ctDNA results than most clinicians realize. A pan-cancer analysis of 16,812 patients found that CH-origin variants were present in 42.3% of individuals, with DNA repair genes disproportionately represented: hematopoietic clones accounted for 39%, 37.9%, 27.4%, and 20.1% of all reported variants in BRCA2, CHEK2, BRCA1, and ATM, respectively (16). Among CRC patients specifically, approximately 29% carry at least one CH-related mutation detectable in plasma cfDNA. Since these variants can remain in circulation for months after surgery, they risk being classified as tumor-derived MRD when they have nothing to do with residual cancer, potentially triggering unwarranted treatment intensification (15, 16).

The genes most frequently involved in CH include DNMT3A, TET2, ASXL1, TP53, ATM, CHEK2, BRCA1, and BRCA2. Three clinical features raise suspicion that a detected variant is CH-derived rather than tumor-derived: a VAF below 5%, a prior history of cytotoxic chemotherapy, and older patient age (15, 39, 40). The relationship with chemotherapy is particularly important: cytotoxic agents exert selective pressure on hematopoietic stem cells, conferring a survival advantage on CH-bearing clones and causing them to expand progressively (39). CH interference is therefore most problematic precisely when MRD assessment carries the greatest clinical weight, at the completion of adjuvant therapy.

Distinguishing CH from tumor-derived mutations requires matched WBC sequencing. Tumor-informed assays that utilize matched WBC sequencing inherently filter these variants and demonstrate superior specificity. Tumor-agnostic approaches may require additional bioinformatic algorithms to distinguish CH from true ctDNA, and the quality of these algorithms varies considerably across commercial platforms (15, 24).

Practical **Box 1** provides questions that clinicians should ask their laboratory or test manufacturer when ordering ctDNA in clinical practice, specifically to assess how CH is addressed.

Box 1 **Practical questions to ask your laboratory about clonal hematopoiesis filtering.**

1. Does this assay include matched white blood cell (WBC) sequencing to filter clonal hematopoiesis variants?
2. What bioinformatic algorithms are used to distinguish tumor-derived mutations from clonal hematopoiesis, and has this algorithm been independently validated in CRC populations?
3. Which genes are most commonly affected by clonal hematopoiesis in this assay’s experience (e.g., DNMT3A, TET2, ASXL1, TP53, ATM, CHEK2, BRCA1, BRCA2), and how are variants in these genes handled?
4. At what variant allele frequency (VAF) threshold does the assay report results, and how does this threshold affect clonal hematopoiesis detection in patients with low-level CH variants (<5% VAF)?
5. Has the assay been validated specifically in patients with prior chemotherapy exposure, which can clonally expand CH-bearing hematopoietic cells and increase the risk of false-positive ctDNA results?

## 12 Evidence gaps and limitations

### 12.1 Evidence gaps in the current literature

Despite significant progress, several key gaps remain. Platform diversity is a fundamental obstacle: the mechanistic differences among tumor-informed whole-exome panels, fixed-gene mutation sets, and methylation-based assays produce results that cannot be directly compared across studies, and this cross-trial incompatibility complicates evidence synthesis considerably (1, 2).

The predictive value of ctDNA is established, but evidence that ctDNA-based therapeutic decisions improve survival is stage-dependent. Stage II offers extensive randomized evidence supporting ctDNA-guided de-escalation while maintaining long-term outcomes (5, 6). In stage III, randomized data show a substantial prognostic difference, but intensifying chemotherapy based on ctDNA positivity has not improved outcomes, and de-escalation strategies fell below the noninferiority margin for standard treatment (7).

False negatives remain clinically significant, particularly in low-volume disease and recurrence patterns where ctDNA detectability is reduced, such as lung-dominant and peritoneal disease with specific plasma-only assays (12), and in settings where guideline-directed imaging already effectively detects recurrences (18). Network meta-analysis data indicate that ctDNA testing after adjuvant therapy completion is the most effective timing strategy for identifying high-risk patients (pooled HR 7.95, 95% CI 5.30-11.91), suggesting residual disease at that point, though optimal surveillance intervals remain under investigation (34).

Patients who are consistently ctDNA-positive but imaging-negative represent a particularly difficult clinical picture, with no broadly accepted treatment approach outside of clinical trials (26).

Optimal surveillance intervals for serial ctDNA testing remain undefined. While network meta-analysis suggests post-adjuvant therapy timing is most prognostic, the optimal frequency of surveillance ctDNA draws (e.g., every 3 months vs. every 6 months) and the appropriate duration of surveillance (e.g., 2 years vs. 5 years) have not been systematically evaluated in randomized trials. This gap has important implications for cost-effectiveness, patient burden, and resource allocation across healthcare systems.

### 12.2 Limitations of this review

This narrative review was not designed as a systematic review with meta-analysis, and formal risk-of-bias assessment was not performed for all included studies. The conclusions are based on a selective synthesis of the most clinically relevant literature rather than an exhaustive search of all available data. The rapidly evolving nature of ctDNA assay technology and ongoing trial activity mean that recommendations current at the time of writing may require updating as new evidence emerges. Furthermore, many of the landmark ctDNA trials were conducted predominantly in high-resource academic settings with limited racial and ethnic diversity, and the generalizability of their performance benchmarks to all patient populations remains uncertain (9).

## 13 Future directions

Several research frontiers are likely to reshape ctDNA’s role in CRC over the coming years.

Assay standardization. The current landscape, in which sensitivity, specificity, and positivity thresholds vary considerably across platforms, makes cross-study comparisons difficult and limits the development of universal clinical guidelines (1, 2). The FDA is actively developing a regulatory framework for MRD assay approval, including guidance on analytical validation requirements, reference standard development, and clinical utility endpoints. Several ctDNA assays are currently under FDA Breakthrough Device Designation review for MRD indications in CRC (20, 31).

Active clinical trials. A number of ongoing trials will define ctDNA’s therapeutic role more precisely. CIRCULATE-Japan (GALAXY; UMIN000039205) continues prospective ctDNA surveillance in stage II-IV CRC and has already published prognostic data supporting ctDNA as an MRD marker (37). The PEGASUS trial (NCT03988036) is evaluating ctDNA-guided intensification with atezolizumab in ctDNA-positive stage III colon cancer. The IMPROVE-IT2 trial (NCT04543604) is prospectively assessing ctDNA in non-operative management of rectal cancer, and the DYNAMIC-Rectal trial is evaluating ctDNA-guided decisions in the neoadjuvant rectal setting.

Multi-analyte and fragmentomics approaches. Integration of ctDNA with other circulating biomarkers, including circulating tumor cells, tumor-educated platelets, and multi-analyte panels combining protein markers with molecular signals, may yield higher diagnostic accuracy than any single analyte alone (20, 41). Fragmentomics-based platforms that exploit the distinct cfDNA fragment length signatures of cancer cells are being refined using machine learning tools, with early data demonstrating improved sensitivity in the low-VAF range (13). These approaches have potential to improve detection of low-shedding recurrence patterns such as peritoneal disease, which remains a performance gap in current platforms.

Cost-effectiveness research. The economic value of ctDNA testing in CRC is not yet well defined. A ctDNA-guided strategy that reduces unnecessary adjuvant chemotherapy use, as demonstrated in DYNAMIC, carries both direct cost savings and quality-of-life benefits. However, the cost of serial ctDNA surveillance over multi-year follow-up has not been systematically modeled against outcomes. Cost-effectiveness analyses using real-world claims data and simulation models are needed to inform payer coverage decisions and guide equitable resource allocation (27, 42).

Patient-reported outcomes and shared decision-making. The psychological impact of ctDNA testing, including anxiety related to positive results and false reassurance from negative results, has not been systematically studied. Integration of patient-reported outcome measures into ctDNA surveillance trials is an important patient-centered research priority. Development of shared decision-making tools that communicate probabilistic ctDNA risk data in an accessible, culturally appropriate manner would help patients and clinicians navigate the uncertainty inherent in molecular surveillance, particularly when imaging and ctDNA results diverge.

Multi-cancer early detection. Methylation-based ctDNA platforms originally developed for multi-cancer early detection may eventually be adapted for post-treatment surveillance in CRC and other gastrointestinal malignancies. While their current primary application is population-level screening, their plasma-only design and high specificity make them candidates for integration into post-treatment follow-up pathways, subject to prospective clinical validation (42, 43).

## 14 A practical 2026 workflow for gastroenterology practice

**Figure 3:**
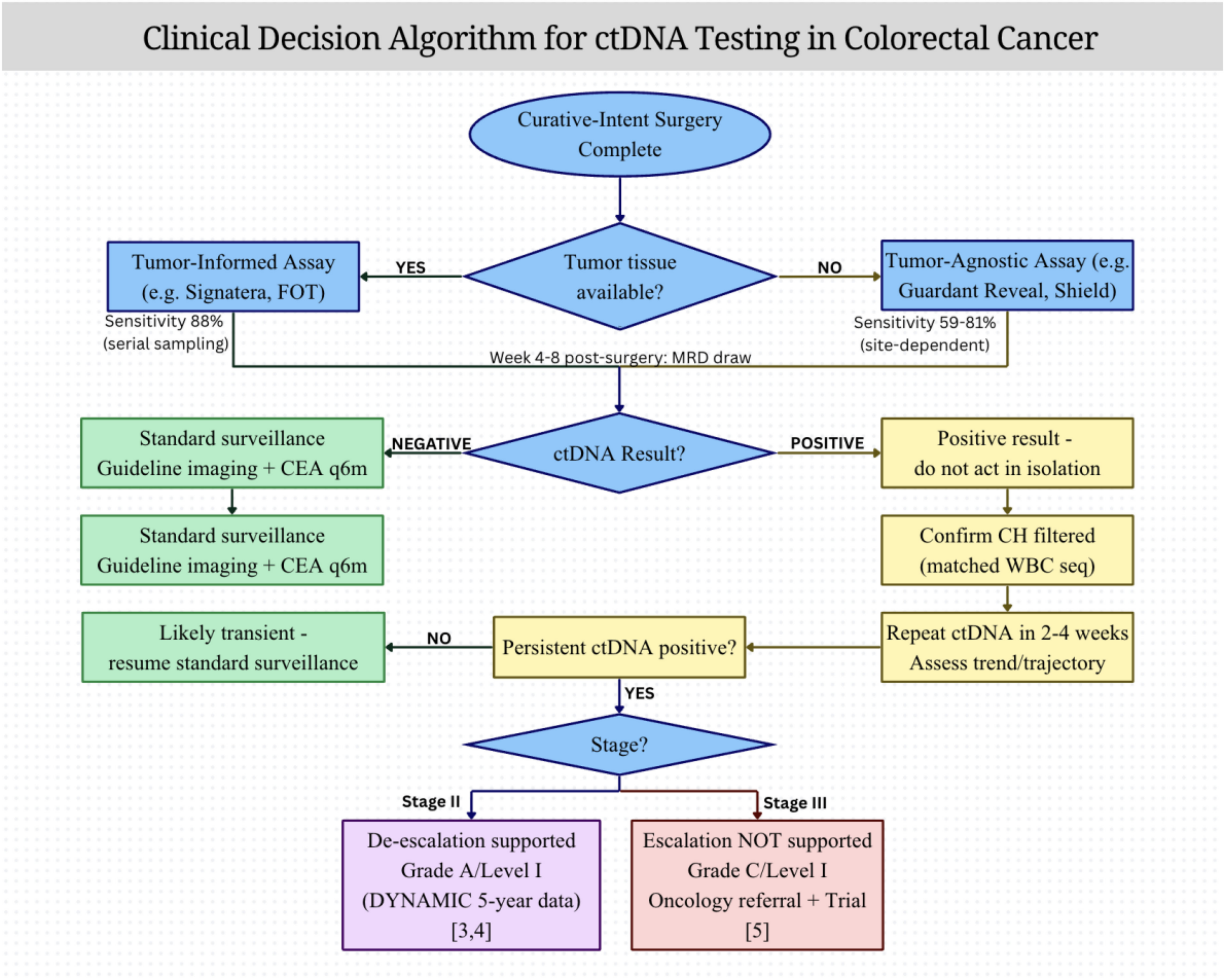
Clinical decision algorithm for ctDNA testing in colorectal cancer. This flowchart guides clinicians through ctDNA test selection based on clinical stage, tissue availability, and treatment intent, and provides evidence-based recommendations for interpreting positive and negative results. Decision branches include: (1) Curative-intent surgery complete – tissue available – tumor-informed vs. agnostic selection; (2) Stage II – ctDNA-guided de-escalation pathway (Grade A); (3) Stage III – prognostic classification and multidisciplinary referral (Grade B/C); (4) Positive result – confirm persistence, involve oncology, consider clinical trial enrollment. The algorithm synthesizes evidence from DYNAMIC (5), DYNAMIC-III (7), and assay comparison data (2, 12) into a structured stepwise guide. Adapted from the workflow in **Table 2**. CEA, Carcinoembryonic Antigen; CH, clonal hematopoiesis; ctDNA, circulating tumor DNA; WBC, white blood cell.

The eight-step framework in **Table 2** guides gastroenterologists through ctDNA test ordering, result interpretation, and follow-up planning in a structured, evidence-anchored manner (**Figure 3**).

**Table 2:** Suggested 2026 workflow for ctDNA testing in CRC.

Step	Key question	Practical considerations	Clinical rationale	Evidence	LOE
1	Why is this test being ordered?	Adjuvant decision vs. surveillance vs. metastatic monitoring	ctDNA has the strongest validated role in MRD risk stratification after curative-intent therapy	(1, 5, 7)	I
2	Is the timing appropriate?	Landmark postoperative sampling: 4–8 weeks post-surgery; very early draws (day 3-7) are prognostic but may reflect surgical cfDNA	Timing affects interpretability and clinical actionability	(5, 7, 12)	I
3	Which assay type fits the clinical question?	Tissue availability, turnaround time, costs, and urgency	Tumor-informed prioritizes specificity for MRD; tumor-agnostic prioritizes speed and accessibility	(2, 12)	I/II
4	Does the platform address CH?	Ask about matched WBC sequencing and bioinformatic CH filtering; see Practical Box 1	Without proper filtering, CH variants in DNMT3A, TET2, TP53, ATM, CHEK2, BRCA1/2 can produce false positives	(15, 16)	II
5	Is the result persistent and clinically plausible?	Repeat unexpected positives; prioritize serial trends over isolated values	Persistence or rising ctDNA is far more actionable than a single low-level positive that resolves	(14, 23)	II
6	What action is evidence-supported at this stage?	Stage II: de-escalation supported (Grade A); Stage III: escalation not supported outside trials (Grade C)	Prognostic value is strong, but therapeutic implications differ substantially by stage	(5–7)	I
7	Who needs to be involved now?	Early oncology input; tumor board discussion where available	ctDNA results should guide coordinated multidisciplinary strategy, not isolated clinical decisions	(1, 26)	III
8	How will follow-up proceed?	Serial ctDNA plus guideline-directed imaging and CEA; ctDNA does not replace imaging	Negative ctDNA does not replace imaging; site-specific sensitivity limitations apply to plasma-only assays	(12, 18)	II

ctDNA, circulating tumor DNA; CRC, colorectal cancer; MRD, minimal residual disease; WBC, white blood cell; CH, clonal hematopoiesis; CEA, carcinoembryonic antigen; LOE, Level of Evidence (I, randomized controlled trial; II, prospective cohort study; III, expert consensus or retrospective data).

## 15 Conclusions: matching assay choice to clinical intent

ctDNA has become a central topic in post-treatment CRC management, offering a molecular window into residual disease that conventional staging and surveillance tools cannot provide. However, not all ctDNA assays are equivalent, and not all positive results carry the same clinical meaning. Tumor-informed and tumor-agnostic platforms address distinct clinical challenges, with important tradeoffs in specificity, sensitivity, turnaround time, and site-specific performance (2, 12).

In 2026, optimal ctDNA utilization for the gastroenterologist rests on three practical principles: (1) the assay must align with the clinical question being asked - tumor-informed platforms for MRD-directed adjuvant decisions where tissue is available, and tumor-agnostic platforms when speed or tissue availability dictate; (2) results must be interpreted within the biological and technical context of the platform used, including known limitations around CH, site-specific shedding, pre-analytical variables, and quantitative VAF trajectory rather than binary thresholds; and (3) findings must be integrated into multidisciplinary pathways rather than prompting isolated, unsupported therapeutic decisions - de-escalation is evidence-supported in stage II disease, but escalation in stage III disease outside clinical trials is not (1, 2, 5, 7, 12, 15, 26).

Evidence grading provides a practical anchor for these decisions. ctDNA-guided de-escalation in stage II colon cancer is supported by high-quality randomized evidence and durable long-term outcomes (Grade A; Level I). ctDNA as a prognostic classifier in stage III disease is well established (Grade B; Level I), but chemotherapy escalation based solely on ctDNA positivity is not supported and should not be applied outside of clinical trials (Grade C; Level I). ctDNA surveillance after curative-intent therapy adds prognostic value and a meaningful imaging lead time (Grade B; Level II), but it does not replace guideline-directed imaging.

Several clinical areas warrant continued investigation, including ctDNA in rectal cancer and neoadjuvant therapy settings, the distinct interpretive considerations introduced by MSI-H tumor biology, disparities in ctDNA access across diverse patient populations, and the unresolved question of optimal surveillance intervals. As clinical evidence matures, assay standardization improves, and cost-effectiveness data emerge, ctDNA testing has the genuine potential to become a routine, outcome-improving component of the gastroenterologist’s approach to post-treatment CRC management.

## Supplementary material

Supplementary Table 1 Summary of key clinical studies informing ctDNA use in CRC.
