# Supplementary material for: Circulating tumor DNA in colorectal cancer: assay selection, clinical applications, and practical integration for gastroenterologists
Source: Front Oncol. 2026 Aug 3;16:1894198. doi: 10.3389/fonc.2026.1894198 (PMC13479687; doi:10.3389/fonc.2026.1894198)
Supplement: Supplementary file 1 [file Supplementaryfile1.docx]

# Supplementary Material

**Supplementary Table S1. Summary of key clinical studies informing ctDNA use in CRC.**

| **Trial/Study** | **Design** | **Stage/Setting** | **Key ctDNA Finding** | **Clinical Implication** | **Study Quality** |
| --- | --- | --- | --- | --- | --- |
| **DYNAMIC (5)** | RCT | Stage II colon cancer | Adjuvant chemo reduced 28% to 15%; 2-yr RFS 93.5% vs. 92.4% | Supports ctDNA-guided de-escalation without sacrificing early survival | Low RoB; prospective RCT, blinded, ITT |
| **DYNAMIC 5-yr follow-up (6)** | Long-term RCT follow-up | Stage II colon cancer | 5-yr RFS 88% vs. 87%; OS 93.8% vs. 93.3%; 87.5% ctDNA clearance on treatment | Confirms long-term safety of de-escalation; clearance is prognostic | Low RoB; mature follow-up, ITT |
| **DYNAMIC-III (7)** | Randomized phase 2/3 | Stage III colon cancer | 3-yr RFS: 87% (neg) vs. 49% (pos); escalation did not improve RFS | Strong prognostic classifier; intensifying chemo based on ctDNA alone not justified | Low RoB; adaptive RCT, control arm modification noted |
| **NRG-GI005 (COBRA) (19)** | Phase II/III (halted) | Stage IIA colon cancer | Prespecified endpoint not met; enrollment halted per stopping rules | Assay specificity critical in low-risk populations; trial halt does not negate ctDNA value overall | Stopped early; limited power |
| **Chen et al. 2021 (14)** | Prospective multicenter observational | Stage II-III CRC | Most accurate independent predictor of recurrence; mean imaging lead time 5.01 months | Serial ctDNA has strong surveillance value; day 3-7 positivity predicts high recurrence risk | Level II; large multicenter; no randomization |
| **Nakamura et al. 2024 (12)** | Prospective (tissue-free epigenomic assay) | Stage II+ CRC (postoperative) | 81% longitudinal sensitivity; 98.2% specificity; 5.3-month lead time; liver 100% vs. peritoneal 40% | Tissue-free epigenomic MRD assay is clinically viable; site sensitivity gaps require supplemental imaging | Level II; single platform; limited diversity |
| **Camblor et al. 2026 (4)** | Systematic review and meta-analysis | Resected CRC (all stages) | Tumor-informed serial sensitivity 88% vs. agnostic 59% (p=0.001) | Assay type significantly affects surveillance performance; tumor-informed preferred for serial MRD surveillance | Level I evidence synthesis; heterogeneity present |
| **Magee et al. 2025 (35)** | Large-scale cohort (16,812 patients) | Advanced cancer (pan-tumor, incl. CRC) | 42.3% harbor CH variants; 29% of CRC patients have CH in plasma cfDNA | CH is highly prevalent; matched WBC sequencing essential to prevent false-positive MRD calls | Level II; large sample; retrospective; no randomization |

*Abbreviations: CRC = colorectal cancer; ctDNA = Circulating tumor DNA; ITT = intention-to-treat; MRD = minimal residual disease; OS = overall survival; RCT = randomized controlled trial; RFS = recurrence-free survival; RoB = risk of bias; VAF = variant allele frequency.*
